# The interplay between sex, ApoE, and insomnia in Alzheimer's disease pathogenesis

**DOI:** 10.1002/alz70855_105689

**Published:** 2025-12-24

**Authors:** Kenny Vetter, Katherine A Murphy, Yu Hou, Jia Geng, Li Zhu, Rui Zhang, Minghui Wang, Dongming Cai

**Affiliations:** ^1^ University of Minnesota, Minneapolis, MN, USA; ^2^ Icahn School of Medicine at Mount Sinai, New York, NY, USA

## Abstract

**Background:**

This work aims to elucidate the mechanisms underlying insomnia‐induced Alzheimer's disease (AD) in a sex‐ and ApoE genotype‐dependent manner to predict treatment responsiveness. Sex‐based differences contribute to AD, with a higher prevalence among females. Additionally, there are known associations between ApoE genotype and sex in AD prognosis. Furthermore, sleep disorders are a risk factor for AD development and are more common in females than males. This work demonstrates pathway interactions that link insomnia, sex, and ApoE genotype that will facilitate a more targeted approach to treat a specific patient population.

**Methods:**

EMR/EHR data was accessed through The All of Us Research Program. The Religious Orders Study/Memory and Aging Program (ROSMAP) human brain transcriptomics and snRNA‐sequencing data was utilized for mapping hub genes and key regulators related to AD and brain cell‐specific gene expression profiles. Dual orexin receptor antagonists (DORA) were tested in cultured neurons from male and female mice that expressed the human ApoE4 allele (knock‐in at the 5xFAD background). Neuronal activity was measured with the Microelectrode Array system from Axion Biosystems.

**Results:**

AllofUS EMR/EHR data mining showed that insomnia was associated with increased risk of development of AD‐related Dementias (ADRD) in females, but not males. Insomnia was associated with increased risks of developing mild cognitive impairment (MCI) in both sexes. ROSMAP transcriptomic analysis identified orexin receptor 1 (*HCRTR1*), a gene dysregulated in insomnia, as a key driver in the AD brain network. Expression of *HCRTR1* increased in neuronal clusters of ApoE4+ female MCI/AD brains compared to ApoE4 male counterparts. This sex difference was not seen in ApoE3+ MCI/AD brains. Neuronal and microglial co‐culture studies demonstrated that HCRTR1 inhibition with DORA promoted neuronal activities in ApoE4+ female brain cells, but not males.

**Conclusions:**

There are many factors that increase an individual's risk for developing AD, including insomnia, sex, and ApoE genotype. Here we have identified a pathway that may contribute to the interplay among these factors in AD pathogenesis. This study could facilitate a better understanding of sex‐ and ApoE‐specific treatment responsiveness, which guide future precision‐medicine guided therapeutic development in AD.

